# High-risk prostate cancer treated with a stereotactic body radiation therapy boost following pelvic nodal irradiation

**DOI:** 10.3389/fonc.2024.1325200

**Published:** 2024-02-06

**Authors:** Jonathan W. Lischalk, Meredith Akerman, Michael C. Repka, Astrid Sanchez, Christopher Mendez, Vianca F. Santos, Todd Carpenter, David Wise, Anthony Corcoran, Herbert Lepor, Aaron Katz, Jonathan A. Haas

**Affiliations:** ^1^Department of Radiation Oncology, Perlmutter Cancer Center at New York University Langone Hospital - Long Island, New York, NY, United States; ^2^Division of Health Services Research, New York University Long Island School of Medicine, New York University Langone Health, Mineola, NY, United States; ^3^Department of Radiation Oncology, University of North Carolina School of Medicine, Chapel Hill, NC, United States; ^4^Department of Medical Oncology, Perlmutter Cancer Center at New York University Langone Health - Manhattan, New York, NY, United States; ^5^Department of Urology, Perlmutter Cancer Center at New York University Langone Hospital - Long Island, New York, NY, United States; ^6^Department of Urology, Perlmutter Cancer Center at New York University Grossman School of Medicine, New York, NY, United States

**Keywords:** prostate, stereotactic body radiation therapy, high-risk, pelvic nodes, boost

## Abstract

**Purpose:**

Modern literature has demonstrated improvements in long-term biochemical outcomes with the use of prophylactic pelvic nodal irradiation followed by a brachytherapy boost in the management of high-risk prostate cancer. However, this comes at the cost of increased treatment-related toxicity. In this study, we explore the outcomes of the largest cohort to date, which uses a stereotactic body radiation therapy (SBRT) boost following pelvic nodal radiation for exclusively high-risk prostate cancer.

**Methods and materials:**

A large institutional database was interrogated to identify all patients with high-risk clinical node-negative prostate cancer treated with conventionally fractionated radiotherapy to the pelvis followed by a robotic SBRT boost to the prostate and seminal vesicles. The boost was uniformly delivered over three fractions. Toxicity was measured using the Common Terminology Criteria for Adverse Events (CTCAE) version 5.0. Oncologic outcomes were assessed using the Kaplan–Meier method. Cox proportional hazard models were created to evaluate associations between pretreatment characteristics and clinical outcomes.

**Results:**

A total of 440 patients with a median age of 71 years were treated, the majority of whom were diagnosed with a grade group 4 or 5 disease. Pelvic nodal irradiation was delivered at a total dose of 4,500 cGy in 25 fractions, followed by a three-fraction SBRT boost. With an early median follow-up of 2.5 years, the crude incidence of grade 2+ genitourinary (GU) and gastrointestinal (GI) toxicity was 13% and 11%, respectively. Multivariate analysis revealed grade 2+ GU toxicity was associated with older age and a higher American Joint Committee on Cancer (AJCC) stage. Multivariate analysis revealed overall survival was associated with patient age and posttreatment prostate-specific antigen (PSA) nadir.

**Conclusion:**

Utilization of an SBRT boost following pelvic nodal irradiation in the treatment of high-risk prostate cancer is oncologically effective with early follow-up and yields minimal high-grade toxicity. We demonstrate a 5-year freedom from biochemical recurrence (FFBCR) of over 83% with correspondingly limited grade 3+ GU and GI toxicity measured at 3.6% and 1.6%, respectively. Long-term follow-up is required to evaluate oncologic outcomes and late toxicity.

## Introduction

Modern radiation oncology improvement efforts in localized high-risk prostate cancer have focused predominantly on three domains: (1) inclusion and duration of novel androgen deprivation therapy (ADT), (2) management of the pelvic lymph nodes, and (3) dose escalation to gross disease. In the two decades since the initial publication of the seminal RTOG 9413 study, arguably no debate has raged hotter in the annals of radiation oncology literature than elective treatment of pelvic lymph nodes in patients with clinically localized prostate cancer (1). Though the topic has prompted scathing editorials and blistering rebuttals, the recent publication of the POP-RT study has convinced many practitioners to adopt elective pelvic radiotherapy as standard practice for patients with high-risk disease at a sufficiently elevated risk of occult nodal involvement (2). While no difference in overall survival (OS) was appreciated at 5 years, significant improvements in biochemical disease-free survival (bDFS) and distant metastasis-free survival (DMFS) were observed, though at the cost of increased treatment-related toxicity. Ultimately, the answer to the benefit of nodal irradiation may not be answered until the publication of three ongoing randomized trials—RTOG 0924 (ClinicalTrials.gov identifier: NCT01368588), PivotalBoost (ISRCTN80146950), and GETUG-AFU-23 (ClinicalTrials.gov identifier: NCT01952223).

According to the National Comprehensive Cancer Network (NCCN) risk stratification system for patients with high-risk diseases, the optimal paradigm for patients treated with radiotherapy is yet undefined. While most practitioners agree that a long-term ADT of at least 18 months is indicated, beyond this common ground, there is limited consensus. At the core of this debate is a randomized study Androgen Suppression Combined with Elective Nodal and Dose Escalated Radiation Therapy (ASCENDE-RT) comparing conventionally fractionated pelvic radiotherapy followed by an external beam radiation therapy (EBRT) versus low-dose rate (LDR) brachytherapy boost to the prostate (3). This trial demonstrated a substantial biochemical progression-free survival (bPFS) benefit for patients receiving a brachytherapy boost, though at 15 years, improvements in OS, prostate-cancer-specific survival (PCSS), and DMFS were not observed. Furthermore, treatment-related toxicity was significantly worse in the brachytherapy arm, with increased rates of cumulative grade 3 genitourinary (GU) toxicity, pad usage, and late catheterization observed at 5 years posttreatment (4, 5).

For patients with low- and intermediate-risk disease, SBRT is now considered a standard and NCCN-sanctioned treatment option given a plethora of data demonstrating excellent oncologic and quality-of-life (QoL) outcomes, including phase III randomized data (6–9). Initially limited to a few centers with specialized expertise, rapid adoption of this approach in the USA has drastically increased accessibility for patients (10, 11). Stereotactic body radiotherapy is particularly attractive as it offers improved convenience to more protracted fractionation schemas, a strong underlying radiobiological rationale, and a toxicity profile that compares favorably to brachytherapy (8, 12). Given these trends in the low- and intermediate-risk cohort, considerable interest has arisen in the use of SBRT as a boost in conjunction with conventionally fractionated pelvic radiotherapy, as in the ASCENDE-RT trial with SBRT replacing the brachytherapy boost (13–18). Moreover, the SBRT technique is not hindered by operative limitations and allows more effective delivery of tumoricidal dose to areas of extracapsular extension and seminal vesicle invasion, features more commonly seen in the high-risk cohort. Preliminary studies have suggested this approach compares favorably to a brachytherapy boost from a QoL standpoint, with the existing literature suggesting low rates of late grade 3+ toxicity (16, 18–20).

We report the early clinical outcomes from the single largest cohort of patients diagnosed with high-risk adenocarcinoma of the prostate treated with conventionally fractionated pelvic nodal irradiation followed by an SBRT boost to the prostate and seminal vesicles.

## Materials and methods

### Patient eligibility

The local Institutional Review Board (Study No. 00001269) approved this single institutional review of patients treated with SBRT for high-risk prostate cancer. All patients were evaluated by a radiation oncologist and deemed appropriate for definitive pelvic nodal irradiation followed by an SBRT boost. All patients underwent pretreatment diagnostic tests, including clinical examination, PSA, and biopsy. Patients were categorized into standard NCCN risk group classifications. Similarly, all patients had standard NCCN-recommended distant staging performed utilizing CT and bone scans, the standard of care at the time of treatment. All patients underwent fiducial marker placement in the prostate approximately 1 week prior to simulation. Fiducial markers were utilized for inter- and intrafractional image guidance. Of note, these patients were predominately treated prior to the routine use of rectal spacer placement. There were no patients with node-positive disease included in this analysis.

### Simulation, planning, and treatment delivery

All patients underwent computed tomography (CT)-based radiation treatment planning simulation (GE Optima 580). An MRI of the prostate was also obtained in the majority of cases at the time of prostate boost simulation and fused with the primary simulation CT scan at the level of the fiducials to assist in target volume delineation. Patients were recommended enema usage prior to simulation and delivery of each SBRT fraction. Nodal radiation was incorporated for all patients with target volume contours generated using standard definitions. Organs at risk (OAR) were contoured and included the rectosigmoid, bladder, penile bulb, small bowel, and femoral heads. Patients were treated using the RTOG pelvic nodal atlas of the time, superiorly starting treatment at the bifurcation of the common iliacs.

All patients underwent pelvic nodal irradiation to a total dose of 4,500 cGy in 25 fractions utilizing either 3D or IMRT technique, which included the prostate and seminal vesicles. If there was evidence of seminal invasion on MRI, the seminal vesicles were included in their entirety. For those without invasion into the seminal vesicles, the proximal 1 cm was included in the prostate clinical target volume (CTV). The prostate target volume (PTV) margins utilized for nodal treatment were 6 mm isometrically. For the SBRT boost portion of treatment, CTV included the entire prostate and proximal or entire seminal vesicles, as described above. A 5-mm isometric expansion of the CTV with a tighter 3-mm posterior margin was used to create the SBRT PTV. A strong recommendation for ADT was made for those medically eligible as a standard practice for men at our institution diagnosed with high-risk prostate cancer. Patients who did not undergo ADT had either medical comorbidities precluding them from ADT administration or declined ADT as a component of their treatment. Dose calculations and planning optimization were performed using Accuray MultiPlan software. Dosimetric constraints for the aforementioned normal structures were utilized based on institutional standards. The dosimetric parameters utilized are included within the **Supplementary Materials**. Of note, uniform implementation of urethral dose constraints was not utilized during this era of treatment. All patients were treated using SBRT delivered over three treatment fractions. Treatments were delivered using a robotic radiosurgical platform with prostate motion accounted for in the *x*-, *y*-, and *z*-planes.

### Follow-up and statistical analysis

Toxicity was retrospectively reviewed using CTACE version 5.0. Patients were followed up using serial PSA and clinical examination typically at 3- to 6-month intervals. Biochemical progression was defined in accordance with the Phoenix definition. Toxicity was measured from the completion of SBRT. Descriptive statistics (median and interquartile range (IQR) for continuous variables; frequency and percent for categorical data) were calculated for the overall sample. Analysis of oncologic outcomes was performed using the Kaplan–Meier method and included the following: OS, disease-free survival (DFS), and 5-year freedom from biochemical recurrence (FFBCR). For FFBCR, death was treated as a censored event. The analyses were accomplished by applying standard methods of survival analysis, that is, computing the Kaplan–Meier product limit curves, where the data were stratified by different groups (i.e., SBRT total dose, age category, PSA category, grade group, and ADT) (21). The PSA nadir was defined as the absolute lowest posttreatment PSA level, regardless of administration of ADT.

In cases where the endpoint event had not yet occurred, the number of years until the last follow-up was used and considered censored. The groups were compared using the log-rank test. The median rates for each group were obtained from the Kaplan–Meier/product-limit estimates, and their corresponding 95% confidence intervals were computed using Greenwood’s formula to calculate the standard error. Univariate and multivariate proportional hazards (Cox) regression was carried out to determine which risk factors were associated with each of the measured outcomes. Data are presented as hazard ratios with their corresponding 95% confidence intervals. A result was considered statistically significant at the *p* < 0.05 level of significance. All analyses were performed using SAS version 9.4 (SAS Institute Inc., Cary, NC, USA).

## Results

### Patient, tumor, and treatment characteristics

From May 2006 to May 2020, a total of 440 patients with a median age of 71 were identified who underwent nodal irradiation to a total dose of 4,500 cGy in 25 daily fractions followed by an SBRT boost to the prostate and seminal vesicles. All patients were diagnosed with high-risk adenocarcinoma of the prostate with a median pretreatment PSA of 11.5 ng/mL (IQR: 6.5 to 24.4 ng/mL). The vast majority of patients were diagnosed with grade groups 4 and 5 disease (*n* = 229 + 131, 82%). The distribution of grade group disease was as follows: grade group 1 (*n* = 9, 2.0%), grade group 2 (*n* = 32, 7.3%), grade group 3 (*n* = 38, 8.6%), grade group 4 (*n* = 229, 52.0%), and grade group 5 (*n* = 131, 29.8%). The majority of patients (*n* = 369, 83.9%) received ADT as a component of treatment. The SBRT boost dose was delivered in three daily fractions, most commonly to a total dose of 2,100 cGy (*n* = 355, 80.7%). The SBRT boost dose was delivered to the remaining patients with a total dose of 1,950 cGy (*n* = 72, 16.4%) and 1,800 cGy (*n* = 12, 2.7%) all in three fractions. Patients treated had a median boost CTV of 59.3 cm^3^ (IQR: 44.8 to 82.6 cm^3^) and were prescribed a median isodose line of 84% (IQR: 83% to 85%). Detailed patient, tumor, and treatment characteristics are listed in **Table 1**.

**Table 1:** Patient, tumor, and treatment characteristics.

	*n*	%
**Age (range)**	Median age 71 [range: 66–76]	
* <60*	38	9%
* [60–70)*	143	32%
* ≥70*	259	59%
**PSA (mg/mL)**	Median PSA 11.5 [range: 6.5–24.4]	
* <10*	196	44%
* [10–20)*	96	22%
* ≥20*	148	34%
AJCC eighth edition stage		
* Unknown*	4	1%
* Tx*	12	3%
* T1*	227	51%
* T2*	168	38%
* T3/T4*	29	7%
Grade group		
* 1*	9	2%
* 2*	32	7%
* 3*	38	9%
* 4*	229	52%
* 5*	131	30%
* Unknown*	1	<1%
ADT		
* Yes*	369	84%
* No*	71	16%
SBRT boost dose (cGy)		
Unknown^*^	1	<1%
* 1,800*	12	3%
* 1,950*	72	16%
* 2,100*	355	81%
**Median boost CTV (cm^3^)**	59.3 [range: 44.8–82.6]	
**Median isodose line (%)**	84 [range: 83–85]	
**Median boost treatment duration (days)**	3	

### Toxicity outcomes

**Figure 1:**
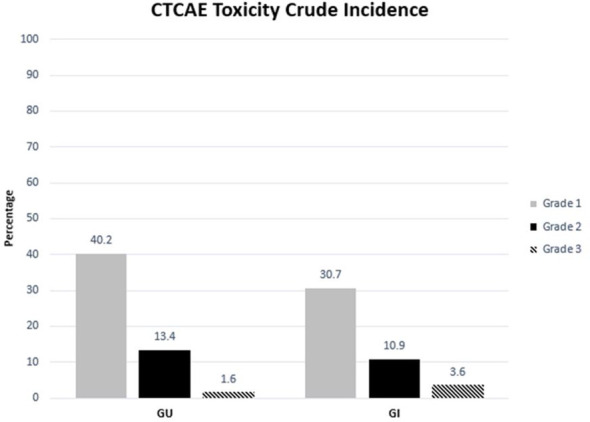
Crude incidence of genitourinary and gastrointestinal toxicity categorized by CTCAE grades 1, 2, and 3.

**Figure 2:**
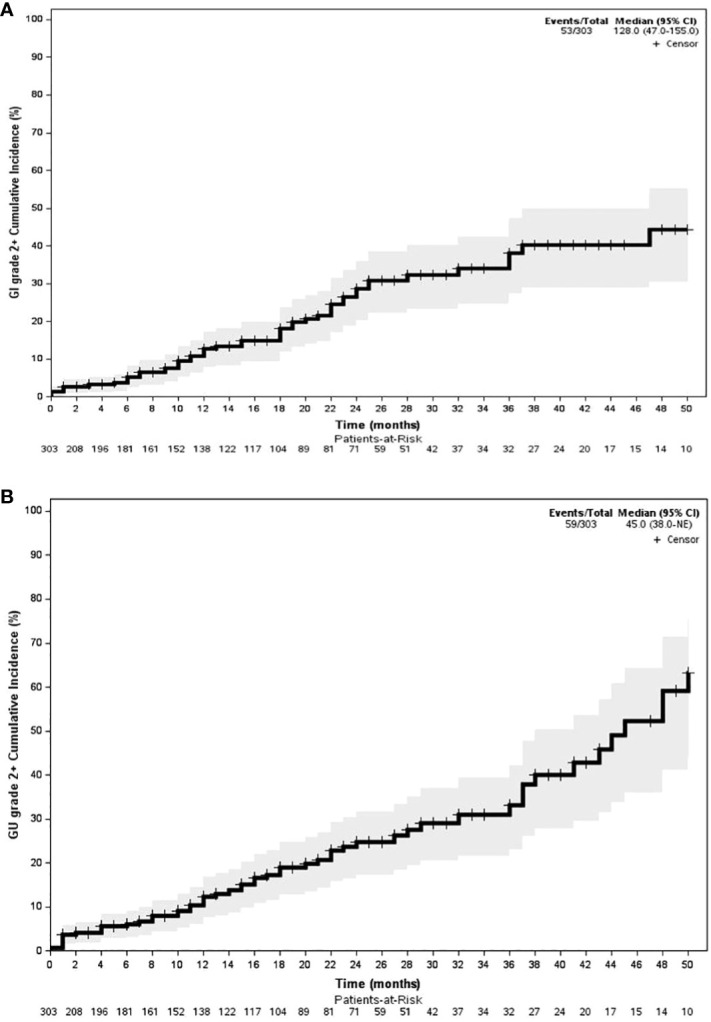
**(A)** GI grade 2+ actuarial analysis. **(B)** GU grade 2+ actuarial analysis.

With an early median follow-up of 2.5 years, the crude incidence of grade 3+ GU and gastrointestinal (GI) toxicity was 1.6% and 3.6%, respectively. All high-grade toxicities were categorized as late toxicities, with the median time to high-grade GU and GI toxicity being 17 and 19 months, respectively. The most common high-grade GU and GI toxicities were urinary tract obstruction and proctitis, respectively. The crude incidence of lower-grade (i.e., grade 2+) GU and GI toxicity was 13.4% and 10.9%, respectively. Similarly, lower-grade toxicities predominately occurred late with the median time to grade 2+ GU and GI toxicity being 15 and 17 months, respectively. The most common grade 1+ GU toxicities included urinary frequency (*n* = 109, 24.8%), urinary tract obstruction (*n* = 29, 6.6%), and dysuria (*n* = 21, 4.8%). The most common grade 1+ GI toxicities included proctitis (*n* = 44, 10%), rectal bleeding (*n* = 32, 7.3%), and diarrhea (*n* = 30, 6.8%). The median follow-up for toxicity was 10 months. Toxicity details are illustrated in **Figures 1**, **2**, and specific graded toxicities are listed in **Supplementary Table S1**.

Univariate (UVA) and multivariate (MVA) analyses were performed to determine associations between demographic and treatment-related factors and ultimate toxicity outcomes (**Tables 2A**, **B**). Grade 2+ GU toxicity was significantly associated with patient older age (HR: 1.05, 95% CI: 1.01–1.09, *p* = 0.02) and AJCC stages T3–4 disease (HR: 3.38, 95% CI: 1.35–8.49, *p* = 0.01) on univariate analysis. This significant association was confirmed on multivariate analysis for both patient age (HR: 1.05, 95% CI: 1.01–1.09, *p* = 0.03) and tumor stage (HR: 3.3, 95% CI: 1.31–8.42, *p* = 0.0115). With respect to grade 2+ GI toxicity, there were no statistically significant predictors of toxicity on univariate analysis. There was a trend toward lower grade 2+ GI toxicity for patients with lower AJCC-staged tumors, though this was not statistically significant. Grade 3+ GU toxicity was significantly associated with grade group 5 (HR: 9.67, 95% CI: 1.07–87.36, *p* = 0.04) and AJCC stages T3–4 disease (HR: 8.33, 95% CI: 1.13–61.59, *p* = 0.04) on univariate analysis (**Supplementary Materials**). Given that the number of subjects who experienced high-grade GI toxicity was so small, logistic regression could not be performed.

**Table 2A:** Univariable and multivariable Cox proportional hazards model analysis of grade 2 or higher genitourinary toxicity.

Grade 2 or higher GU toxicity		Univariate OR	95% CI		*p*-value	Multivariate OR	95% CI		*p*-value
**Age**	*unit = 1*	1.05	1.01	1.09	0.0229^*^	1.05	1.01	1.09	0.0278^*^
**Pretreatment PSA**	*unit = 1*	1.00	1.00	1.01	0.1651				
**Pretreatment PSA as a discrete variable**	*<10*	1.45	0.71	2.95	0.2874				
	*10–20*	1.07	0.44	2.62	0.7728				
	*20+*	*ref*	*ref*	*ref*					
**AJCC seventh edition staging as a discrete variable**	*T1*	*ref*	*ref*	*ref*		*ref*	*ref*	*ref*	
	*T2*	0.87	0.44	1.72	0.6886	0.84	0.42	1.68	0.6300
	*T3/T4*	3.38	1.35	8.49	0.0096^*^	3.32	1.31	8.42	0.0115^*^
**Grade group**	*1–3*	*ref*	*ref*	*ref*					
	*4–5*	1.25	0.54	2.91	0.6049				
**ADT use**	*No vs. yes*	1.30	0.60	2.83	0.5049				
**SBRT boost dose**	*1,800*	3.15	0.81	12.19	0.1518				
	*1,950*	1.35	0.62	2.95	0.5804				
	*2,100*	*ref*	*ref*	*ref*					
**SBRT boost dose**	*1,800/1,950*	1.57	0.78	3.19	0.2082				
	*2,100*	*ref*	*ref*	*ref*					
**Posttreatment PSA nadir**	*unit=0.1*	0.97	0.93	1.02	0.2796				
**Prostate CTV**	*unit=1*	1.00	0.99	1.01	0.9688				

^*^Statistically significant.

**Table 2B:** Univariable and multivariable Cox proportional hazards model analysis of grade 2 or higher gastrointestinal toxicity.

Grade 2 or higher GI toxicity		Univariate OR	95% CI		*p*-value	Multivariate OR	95% CI		*p*-value
**Age**	*unit = 1*	0.98	0.93	1.02	0.2956	0.98	0.93	1.02	0.2847
**Pretreatment PSA**	*unit = 1*	1.00	0.98	1.01	0.5719				
**Pretreatment PSA as a discrete variable**	*<10*	2.29	0.94	5.57	0.1406				
	*10–20*	1.83	0.64	5.23	0.6595				
	*20+*	*ref*	*ref*	*ref*					
**AJCC seventh edition staging as a discrete variable**	*T1*	*ref*	*ref*	*ref*		*ref*	*ref*	*ref*	
	*T2*	0.44	0.19	1.02	0.0551	0.447	0.195	1.027	0.0578
	*T3/T4*	1.42	0.45	4.44	0.5473	1.454	0.463	4.561	0.5212
**Grade group**	*1–3*	*ref*	*ref*	*ref*					
	*4–5*	2.47	0.74	8.28	0.1426				
**ADT use**	*No vs. yes*	1.33	0.56	3.18	0.5181				
**SBRT boost dose**	*1,800*	2.17	0.45	10.35	0.1680				
	*1,950*	0.47	0.14	1.59	0.1095				
	*2,100*	*ref*	*ref*	*ref*					
**SBRT boost dose**	*1,800/1,950*	0.69	0.26	1.82	0.4495				
	*2,100*	*ref*	*ref*	*ref*					
**Posttreatment PSA nadir**	*unit = 0.1*	0.98	0.94	1.03	0.4620				
**Prostate CTV**	*unit = 1*	1.00	0.99	1.01	0.6235				

### Oncologic outcomes

Overall, with an early median follow-up of 2.5 years, these 440 patients with high-risk disease demonstrated 2- and 5-year FFBCR of 97.6% (95% CI: 96.1, 99.2) and 83.1% (95% CI: 73.7, 92.4), respectively, with a median FFBCR that was not reached. The median DFS for the entire high-risk cohort was 8.5 years (95% CI: 7.0, 10.1). Finally, the 2- and 5-year OS were 97.0% (95% CI: 95.2, 98.7) and 82.9% (95% CI: 75.8, 89.9), respectively, with a median OS estimated at 10.1 years (95% CI: 8.8, *inestimable*).

**Figure 3:**
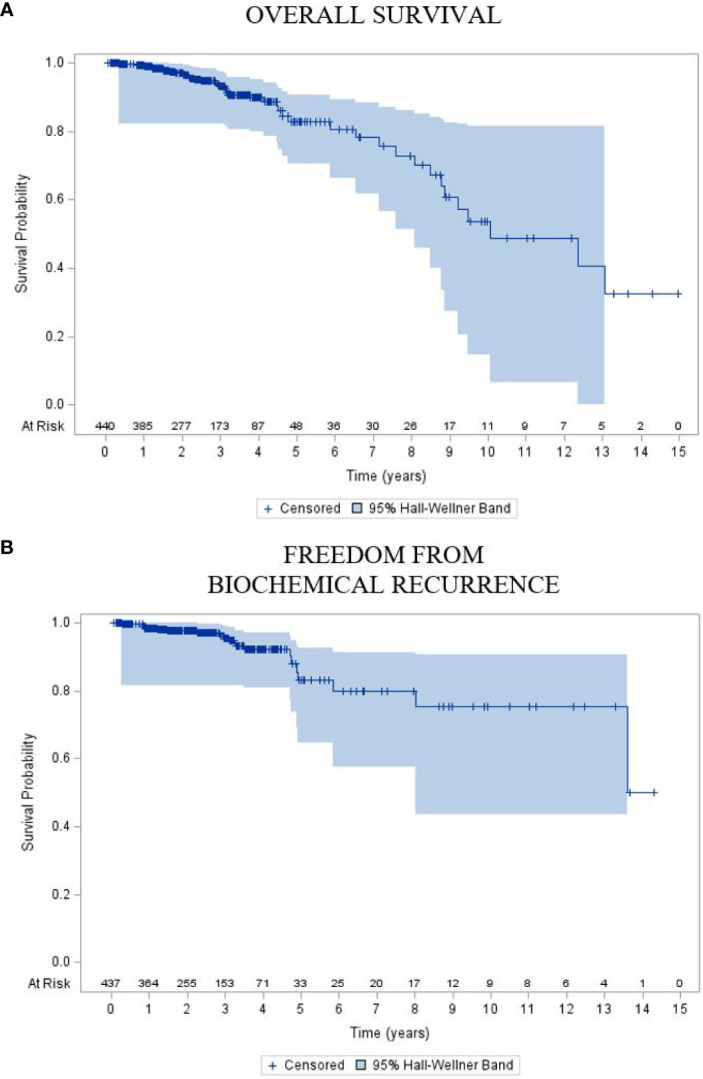
Kaplan–Meier plot of **(A)** overall survival and **(B)** freedom from biochemical recurrence of the entire cohort.

**Figure 4:**
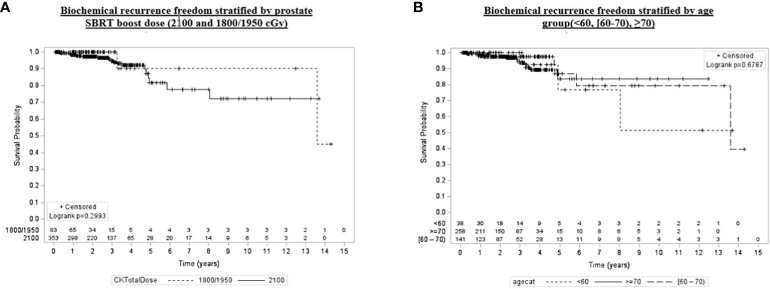
Kaplan–Meier plot of overall biochemical recurrence freedom by prostate SBRT boost dose (2,100 and 1,800/1,950 cGy), age group (<60, [60–70), ≥70), grade group (1–3, 4, 5), and pretreatment PSA (<10, [60–70), ≥20).

**Figure 5:**
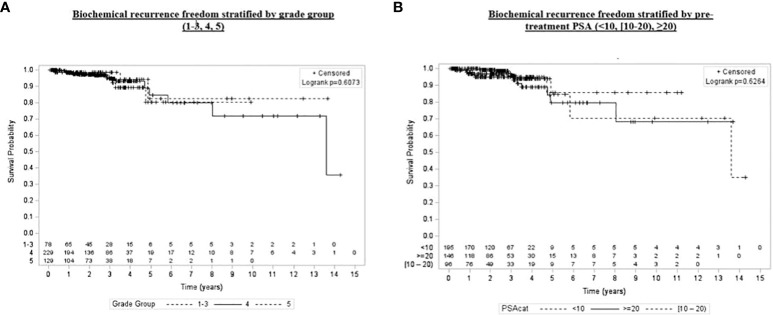
Kaplan–Meier plot of overall biochemical recurrence freedom by grade group (1–3, 4, 5) and pretreatment PSA (<10, [60–70), ≥20).

Univariate and multivariate analyses were performed to determine associations between demographic and treatment-related factors and oncologic outcomes. Overall survival demonstrated an association with both patient age (HR: 1.05, 95% CI: 1.01–1.10, *p* = 0.03) and posttreatment PSA nadir (HR: 1.01, 95% CI: 1.00–1.07, *p* < 0.001) on MVA (**Table 3A**). Freedom from biochemical recurrence was associated with pretreatment PSA (HR: 1.04, 95% CI: 1.00–1.07, *p* = 0.02), posttreatment PSA nadir (HR: 1.00, 95% CI: 1.00–1.01, *p* = 0.04), and AJCC seventh edition staging on MVA (**Table 3B**). Of note, all of the aforementioned significant associations were seen when analyzed as continuous variables, with the exception of stage. There was no association between total radiation boost dose and any oncologic outcome, nor was there any evidence that the prostate cancer grade group was associated with any oncologic outcome on UVA and MVA (**Figures 3**–**5**). Finally, we saw no evidence that lack of ADT usage was related to worse oncologic outcomes; however, the majority of patients in our cohort did receive ADT (84%).

**Table 3A:** Univariable and multivariable Cox proportional hazards models for overall survival.

		Univariate HR	95% CI		*p*-value	Multivariate HR	95% CI		*p*-value
**Age**	*unit = 1*	1.06	1.02	1.10	0.007^*^	1.05	1.01	1.10	0.027^*^
**Pretreatment PSA**	*unit = 1*	0.99	0.98	1.01	0.331				
**Pretreatment PSA as a discrete variable**	*<10*	1.10	0.55	2.23	0.787				
	*10–20*	1.11	0.50	2.45	0.805				
	*20+*	*ref*	*ref*	*ref*					
**AJCC seventh edition staging as a discrete variable**	*T1*	*ref*	*ref*	*ref*					
	*T2*	1.22	0.63	2.34	0.556				
	*T3/T4*	1.73	0.59	5.05	0.315				
**Grade group**	*1*	*ref*	*ref*	*ref*					
	*2*	0.90	0.16	5.18	0.901				
	*3*	0.42	0.06	3.03	0.387				
	*4*	0.67	0.15	2.97	0.597				
	*5*	1.30	0.27	6.19	0.743				
**Grade group**	*1–3*	*ref*	*ref*	*ref*					
	*4–5*	1.16	0.53	2.50	0.714				
**ADT use**	*No vs. yes*	0.87	0.41	1.83	0.705				
**SBRT boost dose**	*1,800*	1.46	0.59	3.64	0.411				
	*1,950*	1.97	0.85	4.53	0.112				
	*2,100*	*ref*	*ref*	*ref*					
**SBRT boost dose**	*1,800/1,950*	1.71	0.88	3.31	0.113				
	*2,100*	*ref*	*ref*	*ref*					
**Posttreatment PSA nadir**	*unit = 0.1*	1.01	1.00	1.01	<0.001^*^	1.01	1.00	1.01	<0.001^*^

^*^Statistically significant.

**Table 3B:** Univariable and multivariable Cox proportional hazards models for biochemical freedom from relapse.

		Univariate HR	95% CI		*p*-value	Multivariate HR	95% CI		*p*-value
**Age**	*unit = 1*	1.03	0.97	1.08	0.362				
**Pretreatment PSA**	*unit = 1*	1.00	1.00	1.01	0.037^*^	1.04	1.00	1.07	0.017^*^
**Pretreatment PSA as a discrete variable**	*<10*	0.64	0.26	1.61	0.345				
	*10–20*	0.76	0.26	2.20	0.616				
	*20+*	*ref*	*ref*	*ref*					
**AJCC seventh edition staging as a discrete variable**	*T1*	*ref*	*ref*	*ref*		*ref*	*ref*	*ref*	
	*T2*	2.47	1.05	5.81	0.038^*^	3.69	1.42	9.58	0.007^*^
	*T3/T4*	0.00	0.00	0.00	0.993	0.00	0.00	0.00	0.990
**Grade group**	*1–3*	*ref*	*ref*	*ref*					
	*4–5*	1.76	0.52	5.93	0.361				
**ADT use**	*No vs. yes*	0.86	0.29	2.54	0.778				
**SBRT boost dose**	*1,800*	0.45	0.05	4.30	0.489				
	*1,950*	0.46	0.06	3.45	0.447				
	*2,100*	*ref*	*ref*	*ref*					
**SBRT boost dose**	*1,800/1,950*	0.45	0.10	2.08	0.309				
	*2,100*	*ref*	*ref*	*ref*					
**Posttreatment PSA nadir**	*unit = 0.1*	1.03	1.01	1.06	0.011^*^	1.00	1.00	1.01	0.038^*^

^*^Statistically significant.

## Discussion

In this single-institutional review of patients with clinically localized high-risk prostate cancer, we report early oncologic and toxicity outcomes that compare favorably to those reported in patients undergoing pelvic nodal treatment followed by a brachytherapy boost or dose-escalated EBRT alone to the prostate. Despite the preponderance of grade groups 4 to 5 disease, an older patient cohort, and an overall high-risk designation, we demonstrate a 5-year FFBCR of over 83%. This was achieved with a very limited risk of high-grade radiation-related GU and GI toxicity (i.e., 1.6% and 3.6%, respectively), which is markedly lower relative to that seen with the use of brachytherapy, albeit with much shorter follow-up, in the ASCENDE-RT trial, where the cumulative incidence of high-grade GU and GI toxicity was 18.4% and 8.1%, respectively. Although there is no clear link between pretreatment factors and GI toxicity, older patient age and higher AJCC stage were associated on MVA with worse grade 2+ GU toxicity, which may be a manifestation of patient baseline urinary function, which is a well-established GU risk factor.

Unsurprisingly, a higher initial PSA at diagnosis was associated with higher rates of biochemical relapse, though without a similar impact on bDFS, DMFS, or OS. Interestingly, a lower PSA nadir was significantly associated with improvements in all measured oncologic outcomes, replicating previous studies that have identified the posttreatment nadir as a meaningful predictive factor. The drop-off in DFS and MFS with long-term follow-up highlights the risk of late failure in this high-risk cohort. These findings underscore the importance of long-term PSA surveillance following treatment, particularly in light of the recent advent of F-18 fluciclovine, Ga-68 PSMA, and F-18 PSMA PET/CT, which boast dramatically improved sensitivity compared to conventional imaging (22). Unfortunately, because of the variety of physicians involved in the delivery of ADT as well as the distance since delivery of that treatment, ADT duration and type were not immediately available for our analysis. Nonetheless, we did not see an association between ADT usage and oncologic outcomes, although this is in part a manifestation of the majority of patients receiving ADT as a component of their treatment. Similarly, there was a range in the total boost dose utilized in three fractions, yet we did not see any association between oncologic outcomes or toxicity and variations in the SBRT total dose. Finally, given the single-arm retrospective nature of the study, it is difficult to comment on survival outcomes.

The rationale for our approach in patients with high-risk disease finds its roots in the ability of this treatment technique to replicate HDR brachytherapy, which was initially established by the seminal work of Fuller et al., published in 2007 (23). The first publication of SBRT used as a boost following nodal irradiation in 2010 comes from the University of California at San Francisco (UCSF) by Jabbari et al., where 38 patients diagnosed with low-, intermediate-, or high-risk prostate cancer were treated with either SBRT monotherapy or nodal irradiation followed by an SBRT boost of 19 Gy in two fractions. Although only half of these patients underwent pelvic nodal irradiation, with an early median follow-up of 18.3 months, there was a 42% rate of grade 2 GU toxicity and an 11% rate of grade 2 GI toxicity (13). A follow-up publication by the same group in 2016 reported the Kaplan–Meier estimate of 5-year relapse-free survival was approximately 83%, with only one grade 3 GU toxicity identified in the cohort of 48 patients (17). Fuller’s “virtual HDR” supposition in 2007 was eventually supported by Chen et al. in 2021, where data demonstrated that SBRT and HDR brachytherapy boosts following IMRT to the pelvic lymph nodes demonstrated strikingly similar oncologic outcomes (18).

Since the initial UCSF publication over a decade ago, there have only been a handful of studies exploring the clinical outcomes of this treatment technique. One of the largest cohorts to date comes from Georgetown University Hospital, where over 100 patients with intermediate- and high-risk disease were treated with conventionally fractionated IMRT to a total dose of 4,500 cGy to 5,040 cGy followed by a robotic SBRT boost of 1,950 cGy in three fractions (16, 19). Approximately half of this group was diagnosed with high-risk disease, and the majority received ADT (63 of 108 patients) as a component of treatment. Paydar et al. reported very modest treatment-related toxicity, with late grade 3 GU toxicity of 6% and late grade 2 GI toxicity of 12%, which is on par with the present study. Ultimately, with early follow-up, the 3-year actuarial biochemical control was 90% for those initially diagnosed with high-risk disease.

In the modern era, two early reports of single-arm prospective trials investigating SBRT boosts have been published and speak to the toxicity analysis measured in the prospective setting. The first comes from France and focuses exclusively on intermediate-risk prostate cancer *sans* pelvic lymph node treatment (24). After an initial course of 4,600 cGy conventionally fractionated radiotherapy to the prostate, a 1,800 cGy in three-fraction boost was applied. The cumulative incidence of GU and GI grade 2 toxicity was quite moderate, measured at 1.4% and 9.3%, respectively, and the 3-year biochemical relapse-free survival was nearly 90%. The second study from Korea explores an SBRT total boost dose question (25). Kim et al. conducted a phase 1/2a trial, termed the ADEBAR study, to prospectively explore the use of pelvic nodal irradiation to 4,400 cGy in 20 fractions randomized to two different SBRT boost regimens of either 1,800 or 2,100 cGy in three fractions. All patients treated on this protocol had either high- or very high-risk prostate cancer and received ADT as a component of their treatment. A total of 26 patients were treated, and at a median early follow-up of 35 months, there were zero acute grade 3+ GU or GI toxicities observed. Late grade 1–2 GU and GI toxicity were measured at 12% and 8%, respectively, which is strikingly similar to the present study of 13.4% and 10.9%, respectively. With early follow-up, 3-year biochemical relapse-free survival was measured at 88.1%. Ultimately, a dose of 2,100 cGy in three fractions was deemed safe and feasible. Similarly, SBRT prescription dose and overall treatment duration were not associated with oncologic outcomes or toxicity rates in the present study.

The future of the prostate SBRT boost lies in its comparative efficacy to moderately hypofractionated EBRT dose escalation or HDR brachytherapy. Several ongoing trials, akin to ASCENDE-RT, directly compare prostate dose escalation in conventionally fractionated IMRT versus an SBRT boost following pelvic nodal irradiation. The HYPOPROST trial (NCT 02300389) aims to directly compare 4,600 cGy in 23 fractions to the whole pelvis, followed by a 1,500 cGy in two-fraction boost to the prostate/seminal vesicles versus a conventionally fractionated boost to 7,600 cGy all in 200 cGy per fraction. One ongoing randomized phase III trial (NCT 01839994) compares dose-escalated IMRT versus IMRT to the pelvic lymph nodes followed by a 2,000 cGy in two fractions of SBRT or HDR brachytherapy boost, both in concert with ADT. Two additional phase II trials (NCT 03380806 and 01985828) are currently recruiting. One trial explores CyberKnife as monotherapy or SBRT boost for intermediate-risk disease, and the other explores the use of a three-fraction SBRT boost (19.5–20 Gy total dose) relative to conventional fractionation following pelvic nodal treatment. Finally, the Francolini et al. trial has demonstrated the efficacy of a moderate hypofractionation technique yielding comparable results for nodal treatment and is naturally analogous to that seen in the prostate alone setting (26). Limitations of the present study include the early follow-up for the disease site as well as its retrospective and single institutional nature, which may limit our ability to evaluate detailed toxicity profiles as well as patient-reported outcomes. Similarly, due to the majority of patients being treated prior to the routine use of a rectal spacer, our study is limited in that we did not control for rectal spacer placement in our analysis.

## Conclusion

We report the largest study of pelvic irradiation with SBRT boost for patients diagnosed with clinically localized high-risk prostate cancer. To date, no study has exclusively reported the outcomes of this technique in an entirely high-risk patient population. We demonstrate a 5-year FFBCR of over 83% with correspondingly limited grade 3+ GU and GI toxicity measured at 3.6% and 1.6%, respectively. Early oncologic and toxicity outcomes compare favorably to those achieved with dose-escalated EBRT alone or in combination with brachytherapy. Prospective randomized data are needed to confirm the safety and efficacy of this approach.

## Data availability statement

Research data are stored in an institutional repository and will be shared upon request with the corresponding author.

## Ethics statement

This single institutional review of patients was approved by the local Institutional Review Board of NYU (Study # 00001269). The studies were conducted in accordance with the local legislation and institutional requirements. Written informed consent for participation was not required from the participants or the participants’ legal guardians/next of kin in accordance with the national legislation and institutional requirements.

## Author contributions

JL: Conceptualization, Investigation, Methodology, Project administration, Supervision, Validation, Writing – original draft, Writing – review & editing. MA: Data curation, Formal analysis, Writing – review & editing. MR: Writing – review & editing. AS: Data curation, Writing – original draft, Writing – review & editing. CM: Data curation, Writing – original draft, Writing – review & editing. VS: Data curation, Writing – review & editing. TC: Writing – review & editing. DW: Writing – review & editing. AC: Writing – review & editing. HL: Writing – review & editing. AK: Writing – review & editing. JH: Conceptualization, Investigation, Methodology, Supervision, Validation, Writing – original draft, Writing – review & editing.

## References

[1] Roach M DeSilvio M Lawton C Uhl V Machtay M Seider MJ. Phase III trial comparing whole-pelvic versus prostate-only radiotherapy and neoadjuvant versus adjuvant combined androgen suppression: Radiation Therapy Oncology Group 9413. J Clin Oncol Off J Am Soc Clin Oncol (2003) 21(10):1904–11. doi: 10.1200/JCO.2003.05.004 12743142

[2] Murthy V Maitre P Kannan S Panigrahi G Krishnatry R Bakshi G. Prostate-only versus whole-pelvic radiation therapy in high-risk and very high-risk prostate cancer (POP-RT): outcomes from phase III randomized controlled trial. J Clin Oncol Off J Am Soc Clin Oncol (2021) 39(11):1234–42. doi: 10.1200/JCO.20.03282 33497252

[3] Morris WJ Tyldesley S Rodda S Halperin R Pai H McKenzie M. Androgen suppression combined with elective nodal and dose escalated radiation therapy (the ASCENDE-RT trial): an analysis of survival endpoints for a randomized trial comparing a low-dose-rate brachytherapy boost to a dose-escalated external beam boost for high- and intermediate-risk prostate cancer. Int J Radiat Oncol Biol Phys (2017) 98(2):275–85. doi: 10.1016/j.ijrobp.2016.11.026 28262473

[4] Rodda S Morris WJ Hamm J Duncan G. ASCENDE-RT: an analysis of health-related quality of life for a randomized trial comparing low-dose-rate brachytherapy boost with dose-escalated external beam boost for high- and intermediate-risk prostate cancer. Int J Radiat Oncol Biol Phys (2017) 98(3):581–9. doi: 10.1016/j.ijrobp.2017.02.027 28581398

[5] Rodda S Tyldesley S Morris WJ Keyes M Halperin R Pai H. ASCENDE-RT: an analysis of treatment-related morbidity for a randomized trial comparing a low-dose-rate brachytherapy boost with a dose-escalated external beam boost for high- and intermediate-risk prostate cancer. Int J Radiat Oncol Biol Phys (2017) 98(2):286–95. doi: 10.1016/j.ijrobp.2017.01.008 28433432

[6] Widmark A Gunnlaugsson A Beckman L Thellenberg-Karlsson C Hoyer M Lagerlund M. Ultra-hypofractionated versus conventionally fractionated radiotherapy for prostate cancer: 5-year outcomes of the HYPO-RT-PC randomised, non-inferiority, phase 3 trial. Lancet Lond Engl (2019) 394(10196):385–95. doi: 10.1016/S0140-6736(19)31131-6 31227373

[7] Brand DH Tree AC Ostler P van der Voet H Loblaw A Chu W. Intensity-modulated fractionated radiotherapy versus stereotactic body radiotherapy for prostate cancer (PACE-B): acute toxicity findings from an international, randomised, open-label, phase 3, non-inferiority trial. Lancet Oncol (2019) 20(11):1531–43. doi: 10.1016/S1470-2045(19)30569-8 PMC6838670 31540791

[8] Kishan AU Dang A Katz AJ Mantz CA Collins SP Aghdam N. Long-term outcomes of stereotactic body radiotherapy for low-risk and intermediate-risk prostate cancer. JAMA Netw Open (2019) 2(2):e188006. doi: 10.1001/jamanetworkopen.2018.8006 30735235 PMC6484596

[9] Jackson WC Silva J Hartman HE Dess RT Kishan AU Beeler WH. Stereotactic body radiation therapy for localized prostate cancer: A systematic review and meta-analysis of over 6,000 patients treated on prospective studies. Int J Radiat Oncol Biol Phys (2019) 104(4):778–89. doi: 10.1016/j.ijrobp.2019.03.051 PMC6770993 30959121

[10] Weiner J P Schwartz D Shao M Osborn V Choi K Schreiber D. Stereotactic radiotherapy of the prostate: fractionation and utilization in the United States. Radiat Oncol J (2017) 35(2):137–43. doi: 10.3857/roj.2017.02026 PMC5518450 28712283

[11] Tang C Lei X Smith GL Pan HY Hoffman KE Kumar R. Influence of geography on prostate cancer treatment. Int J Radiat Oncol Biol Phys (2021) 109(5):1286–95. doi: 10.1016/j.ijrobp.2020.11.055 PMC8647931 33316361

[12] Daşu A. Is the alpha/beta value for prostate tumours low enough to be safely used in clinical trials? Clin Oncol R Coll Radiol G B (2007) 19(5):289–301. doi: 10.1016/j.clon.2007.02.007 17517328

[13] Jabbari S Weinberg VK Kaprealian T Hsu I-C Ma L Chuang C. Stereotactic body radiotherapy as monotherapy or post-external beam radiotherapy boost for prostate cancer: technique, early toxicity, and PSA response. Int J Radiat Oncol Biol Phys (2012) 82(1):228–34. doi: 10.1016/j.ijrobp.2010.10.026 21183287

[14] Lin YW Lin LC Lin KL. The early result of whole pelvic radiotherapy and stereotactic body radiotherapy boost for high-risk localized prostate cancer. Front Oncol (2014) 4:278. doi: 10.3389/fonc.2014.00278 25401085 PMC4215618

[15] Katz A Kang J. Stereotactic body radiotherapy with or without external beam radiation as treatment for organ confined high-risk prostate carcinoma: a six year study. Radiat Oncol Lond Engl (2014) 9:1. doi: 10.1186/1748-717X-9-1 PMC3901326 24382205

[16] Mercado C Kress MA Cyr RA Chen LN Yung TM Bullo EG. Intensity-modulated radiation therapy with stereotactic body radiation therapy boost for unfavorable prostate cancer: the georgetown university experience. Front Oncol (2016) 6:114. doi: 10.3389/fonc.2016.00114 27200300 PMC4858516

[17] Anwar M Weinberg V Seymour Z Hsu IJ Roach M Gottschalk AR. Outcomes of hypofractionated stereotactic body radiotherapy boost for intermediate and high-risk prostate cancer. Radiat Oncol Lond Engl (2016) 11:8. doi: 10.1186/s13014-016-0585-y PMC4721063 26792201

[18] Chen WC Li Y Lazar A Altun A Descovich M Nano T. Stereotactic body radiation therapy and high-dose-rate brachytherapy boost in combination with intensity modulated radiation therapy for localized prostate cancer: A single-institution propensity score matched analysis. Int J Radiat Oncol Biol Phys (2021) 110(2):429–37. doi: 10.1016/j.ijrobp.2020.12.034 33385496

[19] Paydar I Pepin A Cyr RA King J Yung TM Bull EG. Intensity-modulated radiation therapy with stereotactic body radiation therapy boost for unfavorable prostate cancer: A report on 3-year toxicity. Front Oncol (2017) 7:5. doi: 10.3389/fonc.2017.00005 28224113 PMC5293802

[20] Alayed Y Loblaw A Chu W Al-Hanaqta M Chiang A Jain S. Stereotactic body radiation therapy boost for intermediate-risk prostate cancer: A phase 1 dose-escalation study. Int J Radiat Oncol Biol Phys (2019) 104(5):1066–73. doi: 10.1016/j.ijrobp.2019.04.006 31002941

[21] O’quigley J. Statistical methods for survival data analysis. (2nd edition). Elisa Lee, John Wiley, New York, 1992. no. of pages: XII + 482. price: £47.50. ISBN: 0-471-61592-7. Stat Med (1994) 13(8):883–4. doi: 10.1002/sim.4780130812

[22] Hofman MS Lawrentschuk N Francis RJ Tang C Vela I Thomas P. Prostate-specific membrane antigen PET-CT in patients with high-risk prostate cancer before curative-intent surgery or radiotherapy (proPSMA): a prospective, randomised, multicentre study. Lancet Lond Engl (2020) 395(10231):1208–16. doi: 10.1016/S0140-6736(20)30314-7 32209449

[23] Fuller DB Naitoh J Lee C Hardy S Jin H. Virtual HDR CyberKnife treatment for localized prostatic carcinoma: dosimetry comparison with HDR brachytherapy and preliminary clinical observations. Int J Radiat Oncol Biol Phys (2008) 70(5):1588–97. doi: 10.1016/j.ijrobp.2007.11.067 18374232

[24] Pasquier D Peiffert D Nickers P Maingon P Pommier P Lacornerie T. A multicenter phase 2 study of hypofractionated stereostatic boost in intermediate risk prostate carcinoma: A 5-year analysis of the CKNO-PRO trial. Int J Radiat Oncol Biol Phys (2020) 106(1):116–23. doi: 10.1016/j.ijrobp.2019.09.039 31604131

[25] Kim YJ Ahn H Kim CS Kim YS. Phase I/IIa trial of androgen deprivation therapy, external beam radiotherapy, and stereotactic body radiotherapy boost for high-risk prostate cancer (ADEBAR). Radiat Oncol Lond Engl (2020) 15(1):234. doi: 10.1186/s13014-020-01665-6 PMC7542889 33032643

[26] Francolini G Detti B Becherini C Caini S Ingrosso G Di Cataldo V. Toxicity after moderately hypofractionated versus conventionally fractionated prostate radiotherapy: A systematic review and meta-analysis of the current literature. Crit Rev Oncol Hematol (2021) 165:103432. doi: 10.1016/j.critrevonc.2021.103432 34352361

