# Supplementary material for: High-risk prostate cancer treated with a stereotactic body radiation therapy boost following pelvic nodal irradiation
Source: Front Oncol. 2024 Feb 6;14:1325200. doi: 10.3389/fonc.2024.1325200 (PMC10895712; doi:10.3389/fonc.2024.1325200)
Supplement: Supplementary file 1 [file Table_1.docx]

**Supplementary Table 1:** Grade 1, 2, and 3 toxicity incidence stratified by toxicity type

| **Grade 1** | **Count** | **Crude Incidence** |
| --- | --- | --- |
| **Genitourinary** | **177** | **40.2%** |
| URINARY FREQUENCY | 109 | 24.8% |
| URINARY INCONTINENCE | 15 | 3.4% |
| URINARY TRACT OBSTRUCTION | 29 | 6.6% |
| URINARY RETENTION | 5 | 1.1% |
| URINARY URGENCY | 11 | 2.5% |
| UTI | 4 | 0.9% |
| DYSURIA | 21 | 4.8% |
| HEMATURIA | 17 | 3.9% |
| CYSTITIS/CYSTITIS NONINFECTIVE | 4 | 0.9% |
| BLADDER SPASMS | 1 | 0.2% |
|  |  |  |
| **Gastrointestinal** | **135** | **30.7%** |
| RECTAL/ COLONIC HEMORRHAGE | 32 | 7.3% |
| RECTAL FISTULA | 1 | 0.2% |
| RECTAL ULCER | 2 | 0.5% |
| DIARRHEA | 30 | 6.8% |
| PROCTITIS | 44 | 10.0% |
| FECAL INCONTINENCE | 6 | 1.4% |
| ANAL PAIN | 1 | 0.2% |
| HEMORRHOID | 23 | 5.2% |
| CONSTIPATION | 2 | 0.5% |
| ANAL FISSURE | 1 | 0.2% |
| TELANGIECTASIA | 1 | 0.2% |
|  |  |  |
| **Grade 2** |  |  |
| **Genitourinary** | **59** | **13.4%** |
| URINARY FREQUENCY | 28 | 6.4% |
| URINARY INCONTINENCE | 3 | 0.7% |
| URINARY TRACT OBSTRUCTION | 15 | 3.4% |
| URINARY RETENTION | 2 | 0.5% |
| URINARY URGENCY | 0 | 0.0% |
| UTI | 5 | 1.1% |
| DYSURIA | 0 | 0.0% |
| HEMATURIA | 4 | 0.9% |
| CYSTITIS/CYSTITIS NONINFECTIVE | 4 | 0.9% |
| BLADDER SPASMS | 1 | 0.2% |
|  |  |  |
| **Gastrointestinal** | **48** | **10.9%** |
| RECTAL/ COLONIC HEMORRHAGE | 15 | 3.4% |
| RECTAL FISTULA | 1 | 0.2% |
| RECTAL ULCER | 2 | 0.5% |
| DIARRHEA | 3 | 0.7% |
| PROCTITIS | 23 | 5.2% |
| FECAL INCONTINENCE | 0 | 0.0% |
| ANAL PAIN | 0 | 0.0% |
| HEMORRHOID | 4 | 0.9% |
| CONSTIPATION | 0 | 0.0% |
| ANAL FISSURE | 1 | 0.2% |
| TELANGIECTASIA | 0 | 0.0% |
|  |  |  |
| **Grade 3** |  |  |
| **Genitourinary** | **7** | **1.6%** |
| URINARY FREQUENCY | 0 | 0.0% |
| URINARY INCONTINENCE | 0 | 0.0% |
| URINARY TRACT OBSTRUCTION | 3 | 0.7% |
| URINARY RETENTION | 0 | 0.0% |
| URINARY URGENCY | 0 | 0.0% |
| UTI | 0 | 0.0% |
| DYSURIA | 0 | 0.0% |
| HEMATURIA | 2 | 0.5% |
| CYSTITIS/CYSTITIS NONINFECTIVE | 2 | 0.5% |
| BLADDER SPASMS | 0 | 0.0% |
|  |  |  |
| **Gastrointestinal** | **16** | **3.6%** |
| RECTAL/ COLONIC HEMORRHAGE | 6 | 1.4% |
| RECTAL FISTULA | 0 | 0.0% |
| RECTAL ULCER | 2 | 0.5% |
| DIARRHEA | 0 | 0.0% |
| PROCTITIS | 8 | 1.8% |
| FECAL INCONTINENCE | 0 | 0.0% |
| ANAL PAIN | 0 | 0.0% |
| HEMORRHOID | 0 | 0.0% |
| CONSTIPATION | 0 | 0.0% |
| ANAL FISSURE | 0 | 0.0% |
| TELANGIECTASIA | 0 | 0.0% |
|  |  |  |
