# Supplementary material for: High-risk prostate cancer treated with a stereotactic body radiation therapy boost following pelvic nodal irradiation
Source: Front Oncol. 2024 Feb 6;14:1325200. doi: 10.3389/fonc.2024.1325200 (PMC10895712; doi:10.3389/fonc.2024.1325200)
Supplement: Supplementary file 2 [file Table_2.docx]

**Supplementary Table 2:** Dosimetric Constraints for SBRT boost treatment to a toal doe of 21Gy in 3 fractions

| **Structure** | **Dose Limit** |
| --- | --- |
| D95≥21Gy | |
| D0 ≤25.3Gy | |
| Max Dose NOT inside bladder or rectum/bowel | |
|  | |
| Rectum | V22.7Gy < 5% |
|  | V20.3Gy < 20% |
|  | V17.4Gy < 40% |
|  | V11Gy < 60% |
| Bladder | V22.7Gy < 60% |
|  | V10Gy < 50% |
