# Supplementary material for: High-risk prostate cancer treated with a stereotactic body radiation therapy boost following pelvic nodal irradiation
Source: Front Oncol. 2024 Feb 6;14:1325200. doi: 10.3389/fonc.2024.1325200 (PMC10895712; doi:10.3389/fonc.2024.1325200)
Supplement: Supplementary file 3 [file Table_3.docx]

**Supplementary Table 3:** UVA Odds ratios from the Logistic regression models for Grade 3 or higher GU toxicity

| **Grade 3 or higher GU toxicity** |  | **Univariate OR** | **95% CI** | | **p-value** |
| --- | --- | --- | --- | --- | --- |
| **Age** | *unit=1* | 1.01 | 0.90 | 1.13 | 0.8449 |
| **Pretreatment PSA** | *unit=1* | 1.01 | 1.00 | 1.03 | 0.1011 |
| **Pretreatment PSA as a discrete variable** | *<20* | 0.76 | 0.13 | 4.59 | 0.7626 |
|  | *20+* | *ref* | *ref* | *ref* |  |
| **AJCC seventh edition staging as a discrete variable** | *T1* | *ref* | *ref* | *ref* |  |
|  | *T2* | 0.67 | 0.06 | 7.49 | 0.7479 |
|  | *T3/T4* | 8.33 | 1.13 | 61.59 | 0.0377* |
| **Grade group** | *1-4* | *ref* | *ref* | *ref* |  |
|  | *5* | 9.67 | 1.07 | 87.36 | 0.0433* |
| **ADT use** | *No vs. Yes* | 3.54 | 0.58 | 21.56 | 0.1708 |
| **SBRT boost dose** | *1800* |  |  |  |  |
|  | *1950* |  |  |  |  |
|  | *2100* | *ref* | *ref* | *ref* |  |
| **SBRT boost dose** | *1800/1950* |  |  |  |  |
|  | *2100* | *ref* | *ref* | *ref* |  |
| **Post treatment PSA nadir** | *unit=0.1* |  |  |  |  |
| **Prostate CTV** |  |  |  |  |  |

*Statistically Significant
